# Will differentiated care for stable HIV patients reduce healthcare systems costs?

**DOI:** 10.1002/jia2.25541

**Published:** 2020-07-20

**Authors:** Bruce A Larson, Sophie JS Pascoe, Amy Huber, Lawrence C Long, Joshua Murphy, Jacqui Miot, Matthew P Fox, Nicole Fraser‐Hurt, Sydney Rosen

**Affiliations:** ^1^ Department of Global Health Boston University School of Public Health Boston MA USA; ^2^ Health Economics and Epidemiology Research Office Department of Internal Medicine School of Clinical Medicine Faculty of Health Sciences University of the Witwatersrand Johannesburg South Africa; ^3^ Department of Epidemiology Boston University School of Public Health Boston MA USA; ^4^ The World Bank Washington DC USA

**Keywords:** HIV, cost of ART, stable patients, adherence clubs, decentralized medication delivery, adherence guidelines, South Africa

## Abstract

**Introduction:**

South Africa’s National Department of Health launched the National Adherence Guidelines for Chronic Diseases in 2015. These guidelines include adherence clubs (AC) and decentralized medication delivery (DMD) as two differentiated models of care for stable HIV patients on antiretroviral therapy. While the adherence guidelines do not suggest that provider costs (costs to the healthcare system for medications, laboratory tests and visits to clinics or alternative locations) for stable patients in these differentiated models of care will be lower than conventional, clinic‐based care, recent modelling exercises suggest that such differentiated models could substantially reduce provider costs. In the context of continued implementation of the guidelines, we discuss the conditions under which provider costs of care for stable HIV patients could fall, or rise, with AC and DMD models of care in South Africa.

**Discussion:**

In prior studies of HIV care and treatment costs, three main cost categories are antiretroviral medications, laboratory tests and general interaction costs based on encounters with health workers. Stable patients are likely to be on the national first‐line regimen (Tenofovir/Entricitabine/Efavarinz (TDF/FTC/EFV)), so no difference in the costs of medications is expected. Laboratory testing guidelines for stable patients are the same regardless of the model of care, so no difference in laboratory costs is expected as well. Based on existing information regarding the costs of clinic visits, AC visits and DMD drug pickups, we expect that for some clinics, visit costs for DMD or AC models of care could be less, but modestly so, than for conventional, clinic‐based care. For other clinics, however, DMD or AC models could have higher visit costs (see Table 2).

**Conclusions:**

The standard of care for stable patients has already been “differentiated” for years in South Africa, prior to the roll out of the new adherence guidelines. AC and DMD models of care, when implemented as envisioned in the guidelines, are unlikely to generate substantive reductions or increases in provider costs of care.

## 1 INTRODUCTION

Global goals for treatment of HIV include reaching 90% of diagnosed HIV‐positive individuals with antiretroviral therapy (ART) by 2020 and 95% by 2030 [1, 2]. For many low‐ and middle‐income countries, the need to add millions more patients to national treatment programmes poses a substantial challenge in terms of healthcare system capacity and cost [3, 4]. One proposed solution to this problem is known as differentiated care, or differentiated service delivery (DSD), in which delivery of HIV testing, care and treatment is tailored to patients’ needs and health systems’ capacity [5, 6]. Approaches to service delivery might be differentiated, for example by patient subpopulation (pregnant or breastfeeding women, other adult women, men, children, adolescents, high‐risk populations) or by progress along the HIV care and treatment cascade (newly diagnosed, newly initiated, treatment experienced and virally suppressed, failing on treatment, lost to follow‐up) [5]. Among the goals of DSD models are to improve clinical outcomes and to lessen the burden and costs of obtaining treatment for patients.

It is also hoped that DSD models will lessen the burden of HIV care on healthcare facilities, for example by diminishing clinic congestion and reducing costs to the providers of care [5, 7, 8, 9]. As studies have begun to document the effectiveness (clinical outcomes) of DSD model participation for adult patients considered to be “stable” on ART [10, 11, 12, 13, 14], suggestions have been made that differentiated treatment can be “more cost‐effective” than an undifferentiated approach, from the perspective of the provider of care, which is usually the health system [15, 16]. Empirical data to support these claims are scarce, however, in particular due to a lack of primary cost estimates for DSD models (for a recent review, see [17]).

In studies conducted to date [18, 19, 20, 21, 22, 23, 24, 25], outpatient costs of care from a provider’s perspective for HIV patients on ART include four main cost categories: ARV medications; laboratory tests; patient interaction (visit) costs based on encounters with healthcare workers (e.g. nurses, pharmacists) and fixed or overhead costs for infrastructure, administrative staff, etc.).

The focus of this discussion is on DSD models for patients who are already stable on ART (already virally suppressed). For this category of patients, DSD models typically may include one or more of the following changes to standard care: (1) longer medication refills within the prescription period (e.g. three months or even six months dispensed at once, rather than just one month); (2) different locations or procedures for medication collection and/or other services traditionally offered at clinics and (3) different cadres of staff providing services. Adherence Clubs and Decentralized Medication Delivery, discussed in more detail below, are two common DSD models for stable patients that each include at least two of the three changes listed earlier.

Whether costs to health systems will go up or down in response to the widespread advent of DSD models for stable ART patients will largely depend on how differentiated models differ from conventional care, in terms of such characteristics as visit frequency, medication refill mechanism and provider cadre. In this discussion, we use an evaluation of South Africa’s National Adherence Guidelines for Chronic Diseases (HIV, TB and NCDs) [26], referred to here as the AGL evaluation [10, 27], to examine how adherence clubs (AC) and decentralized medication delivery (DMD) for stable patients can affect provider costs.

We use the adherence guidelines to discuss provider costs for patient care if provided according to guidelines (modelled costs). We organize our discussion by posing and addressing eight questions that help to identify the conditions under which provider costs of care for stable HIV patients could fall or rise, compared to the prior standard of care (SOC). Although this discussion addresses DSD models specifically implemented in South Africa as part of the rollout of the national adherence guidelines, the approach followed here can be applied to specific DSD models beyond South Africa.

We do not address changes to patient costs, such as transport fares and lost wages; these are an important aspect of DSD models but have been reviewed recently and are outside the scope of this discussion [28]. Details of the overall evaluation of the National Adherence Guidelines have previously been presented [10, 27].

## 2 DISCUSSION

In this discussion, we pose and answer eight sequential questions relating to the provider costs of DSD models of ART delivery under the South African National Adherence Guidelines. The questions are as follows:

1. What is a stable ART patient?
2. What is the conventional model of care used as a comparison?
3. What are the AC and DMD models of care?
4. Did implementation of these models affect patient outcomes?
5. What do the guidelines say about costs?
6. Were there differences between SOC and the new models in annual costs of antiretroviral medications and laboratory tests?
7. Were there differences in interaction costs, including clinic visits, model interactions and medication pickups?
8. Should differentiated care for stable HIV patients in South Africa increase or decrease costs of HIV care and treatment?

### 2.1 Question 1: What is a stable ART patient?

In South Africa’s National Adherence Guidelines, AC and DMD models of care are intended specifically for stable patients. A stable HIV patient is defined as follows:

- an adult (≥18 years);
- on the same antiretroviral (ARV) regimen for at least 12 months;
- has had a viral load test within the past six months; and
- has had two consecutive undetectable viral loads (at the time of the evaluation was defined as <400 copies/mL).

A stable patient is thus a treatment experienced, virally suppressed adult. These patients have already demonstrated that they can successfully navigate the conventional SOC, which likely required frequent clinic visits and substantial travel and waiting times.

### 2.2 Question 2: What is the conventional model of care used as a comparison?

To some degree, ART delivery in South Africa has been differentiated for different types of patients for years. Guidelines laid out different procedures for patients on first‐ and second‐line regimens; pregnant women and non‐pregnant adults of either sex and advanced disease patients versus those presenting either without illness or with high CD4 counts. At the time the Adherence Guidelines were developed, the SOC for clinic‐based care for stable patients required six clinic visits annually, with up to two‐month dispensing. According to primary healthcare guidelines in South Africa, each of the six annual visits for stable patients was a “full visit.” These visits would include screening for and discussion of symptoms (e.g. for TB and ART side effects); weight measurement; determining pregnancy status for women; a clinical exam for WHO staging; adherence counselling and counselling around lifestyle choices and risk behaviours [29]. At some visits, for example for annual viral load monitoring, a blood sample might also be collected. In this article, “SOC” refers to this pre‐adherence guidelines SOC.

Under the SOC, prescriptions were written for six‐month intervals, but required medication pickups at two‐month intervals. Anecdotal evidence suggests that rather than making every bimonthly visit a “full” visit, clinics might allow short medication pick‐up visits for stable patients (essentially a type of differentiated care) at the second and third visit after each prescription was written (i.e. in any year‐long cycle, visits at months 0 and 6 were full clinic visits; visits at months 2, 4, 8 and 10 could be limited medication pickups with little or no clinician interaction). We note that while medication pickup visits might have been short from the clinic’s perspective, patients often still endured long waits for medication collection, as well as the costs in time and money of clinic transport.

Except in a few pilot projects or studies, under the SOC all services were delivered at established clinics, with public health nurses (the highest ranking nurses in the system) serving as the primary cadre for standard treatment initiation and management [26].

### 2.3 Question 3: What are the AC and DMD models of care?

The Adherence Guidelines included AC and DMD as two DSD models for stable HIV patients. Both were designed to allow stable patients to make fewer visits to clinics annually and to collect their medication in a more convenient and streamlined manner.

ACs are healthcare worker‐led groups of up to 30 stable patients who meet at clinic facilities or other community locations. At AC meetings, patients receive a basic clinical assessment, referral if required, peer support and antiretroviral medications. DMD is an individual model where patients collect medications at designated pickup points rather than in the usual pharmacy queue. A pick‐up point can be in or just outside the clinic or at a separate location external to the clinic, such as a private pharmacy. For both models, medications are often pre‐packaged and delivered through the Central Chronic Medicine Dispensing and Distribution (CCMDD) programme.

For patients enrolled in either AC or DMD models, the four SOC clinic visits at months 2, 4, 8 and 10 are substituted for four AC visits or DMD pickups. Patients pick up two months of medication at each AC or DMD visit and clinic visit. (South Africa, at this time, only allows a maximum of two months’ medication dispensing at a time.)

The AC or DMD models of care for stable patients are expected to provide at least two direct benefits to these patients. First, if AC or DMD clinic visits or medication pickups occur at more convenient locations and/or are less time consuming or possibly less frequent than SOC visits, moving from SOC to AC or DMD could reduce financial and time costs to patients. Second, a more convenient, friendlier or less expensive delivery model could improve or at least maintain adherence to medications over time [15]. If a large enough proportion of patients at clinics were stable, enrolled in either an AC or DMD model, and complied with model guidelines, then clinics could anticipate substantially fewer “full” patient visits per month. In that case, the differentiated models could ease crowding at facilities, lighten the burden on healthcare providers, and reduce waiting times and possibly improve the quality of care for patients remaining in SOC.

### 2.4 Question 4: Did implementation of these models affect patient outcomes?

At the start of the national rollout of the adherence guidelines in 2015, the NDOH identified 24 clinics where implementation would be staggered to allow a cluster‐randomized evaluation of specific interventions called for in the guidelines. For this AGL evaluation, the outcomes of patients in early intervention clinics and enrolled in the interventions were compared to patients who would have been eligible for the interventions, but remained at clinics that still offered only SOC. As reported in Table 1, results for 12‐month viral suppression were similar for both study groups; further details have been reported previously [27].

**Table 1:** Viral suppression in the adherence guidelines evaluation [10]

Outcome	Adherence clubs		Adjusted risk difference (95% CI)	Decentralized medication delivery		Adjusted risk difference (95% CI)
	Standard of care n = 294	Intervention n = 277		Standard of care n = 346	Intervention n = 232	
Known viral suppression at 12 months after model enrolment or eligibility	80%	79.6%	3.8% (−6.9% to 14.4%)	74.3%	77.2%	−1.0% (−12.2% to 10.1%)

### 2.5 Question 5: What do the guidelines say about costs?

The NDOH guidelines address upfront costs to prepare for AGL implementation and other possible recurrent costs linked to implementation. Upfront costs may include capital equipment purchases, but are mainly related to training staff. As long as both training of trainers and training of clinic staff are modest in scale and the trainees continue working and using their new skills, training costs can be amortized over several years and across many patient interactions, and cost per patient should thus be very low. The guidelines explicitly state that additional infrastructure is not required for scaling up implementation of these models of care. The guidelines also state that additional human resources, beyond existing staff, should not be required, although this does assume that facilities are already fully staffed under SOC, which many were not. The guidelines thus do not suggest that the AC or DMD models of care will reduce overall provider costs for stable patients compared to SOC but should not add substantially to those costs either.

### 2.6 Question 6: Were there differences between SOC and the new models in annual costs of antiretroviral medications and laboratory tests?

Costs of patient care from the provider’s perspective depend on the resources/inputs provided for that care. Prior studies of the outpatient cost of care for HIV patients on ART define four main cost categories: ARV medications; laboratory tests; patient interaction (visit) costs based on encounters with healthcare workers (e.g. nurses, pharmacists) and fixed or overhead costs for infrastructure, administrative staff, etc., which tend to be very modest [18, 19, 20, 21, 22, 23, 24, 25].

As noted earlier, the AC and DMD models were limited to stable patients, medications and laboratory tests followed national ART guidelines regardless of the service delivery model, and retention in care was similar between SOC and the new models [27]. We might thus expect no differences in the costs of ARV medications or laboratory tests. Here we look first at medication costs, then laboratory tests.

In South Africa during the rollout of the adherence guidelines, TDF/FTC/EFV was the standard, first‐line ARV regimen provided as a once‐per day, fixed‐dose combination. Patient‐level data from the AGL evaluation confirms that essentially all patients included in the AC and DMD study groups (comparison and intervention) received the fixed‐dose combination of TDF/FTC/EFV, and at least 96% of all drug pickups in each cohort over the one‐year study follow‐up period were TDF/FTC/EFV. The cost for 12 months of the standard regimen was $119 using the 2017 average annual exchange rate (13.33 ZAR/US$) [30, 31].

Laboratory testing guidelines for stable patients also do not vary by model of care, and laboratory test costs would thus also be expected to be identical for stable patients regardless of their model of care. For stable patients, guidelines call for an annual viral load for all patients and an annual creatinine test for patients on TDF. In the AGL evaluation, which had a one‐year follow‐up period, most patients in all study groups received one viral load test (the 0.8 tests on average for the AC intervention and comparison groups and 0.9 tests for the DMD intervention and comparison groups). Creatinine tests were less common (on average between 0.3 and 0.5 tests per group. The NHLS fee for a viral load test was $24.09 per test in 2017, and the fee for a creatinine test was $2.15 [32]. Few other tests were performed for patients in either group, with no substantial differences across groups.

### 2.7 Question 7: Were there differences in interaction costs?

As explained earlier, the main opportunity for cost differences between SOC and the new differentiated models was in interaction costs (i.e. clinic and medication pickup visits). The SOC at the time of our evaluation required six “full” visits per year. Long *et al*. [25] estimated an average cost of ZAR 86.64 for staff salaries for a clinic visit for an HIV patient on ART at a primary health clinic in South Africa in 2014, which was the equivalent of $7.73 in 2017 [31, 33]. A very similar cost of $8.17 for a follow‐up clinic visit for chronic care (pre‐exposure prophylaxis patients in this case) in 2017/2018 is reported in [34]. Shorter visits to collect ARV medications – not sanctioned by guidelines but certainly allowed in practice – likely cost less. In the analysis reported by Long *et al*. [24], the cost of a clinic visit to primary healthcare nurse was estimated at $2.73, whereas those limited to a pharmacy assistant was estimated to cost $1.59 [31, 33].

The CCMDD programme is the main mechanism for implementing the DMD model of care for stable HIV patients. CCMDD charges a fee per month of ARVs provided to a DMD pickup point. If two months of ARVs are collected, then the CCMDD fee is twice the per‐month fee. For this discussion, we use the average fee charged by CCMDD service providers to deliver two months of ARV medications (ZAR 23.90 per month collected in 2017, equal to $3.59 for a two‐month refill). If the clinic must provide extra support, for example for preparing and submitting electronic scripts, correcting incorrect scripts, and monitoring whether CCMDD registered patients have collected their medication from a pick‐up point, then the cost might increase. For example if such activities were completed by a PHC nurse and required a similar level of effort as with a clinic visit ($2.73 as noted earlier, then a DMD pickup cost would increase to $6.32 (an interaction with a PHC nurse and the CCMDD delivery fee).

A growing literature describes experiences with implementing ACs [11, 12, 14, 35, 36, 37, 38, 39, 40] and documents wide variation in AC implementation. The staff involved (public sector staff or NGO staff, lay or clinical cadres), location (at a clinic, next to a clinic, in a community location a distance from a clinic), types of patients served by the club (only adults, only adult and stable patients, other NCD patients) and numbers enrolled per club (up to 30 in the guidelines but often many fewer or more) – all vary widely. As a result, the cost per patient visit to an AC is likely to vary widely as well, both across clinics and between clubs at the same clinic. In addition, ACs may receive ARV medications through the CCMDD delivery mechanism, which would then incur a CCMDD delivery fee along with the cost for an AC visit.

We have found only one published report of the cost for an AC visit [15]. Table 2 shows how the estimate reported by Bango *et al*. [15] was converted into an AC visit cost of $6.67 for this discussion. We include in this discussion a range from $3.34, allowing for an AC visit cost that is 50% lower than in Bango *et al*., to $10.01, an AC visit cost that is 50% higher. Costs could be lower or higher than the base case due, for example to larger or smaller group sizes and/or less or more health worker time per AC group visit.

**Table 2:** Unit cost for adherence club (AC) visit (US $2017)

Value	AC visit cost	Clinic visit	Notes
Visit cost inflation adjusted to 2017 and converted to US dollars ($)	$7.61	$8.67	Both costs from [15]. Excludes overhead costs. 40% cumulative inflation between 2011 and 2017; 2017 exchange rate of ZAR/$ = 13.33 [31, 33]
Cost difference AC – clinic visit	−$1.06		This difference is used in the following row to estimate a base case AC visit cost for comparison a base case clinic visit cost
Base case visit cost	$6.67	$7.73	Source for a primary healthcare clinic visit [25] also inflation adjusted to 2017 and converted to $; AC cost = 7.73 to $1.06
AC visit cost 50% lower	$3.34		For sensitivity analysis
AC visit cost 50% higher	$10.01		For sensitivity analysis

Table 3 presents the anticipated frequency of visits of various types and the projected annual costs for all visits per year. Visit costs/patient/year range from a low of $26 for SOC with intermediate visits conducted by nurses and limited to medication pickups, to a high of $56 if AC visits are assumed to cost 50% more than the base case. There is no clear trend in visits costs by model, suggesting that the real cost will depend heavily on the details of implementation in a particular site or programme.

**Table 3:** Unit costs (cost per visit) and annual cost for all visits ($2017)

Visit type	Cost/visit (from Table 2)	Expected numbers of visits^a^				Annual visit costs
		Full clinic visits	Short clinic visits	Decentralized medication delivery (DMD) visits	Adherence club (AC) visits	(US$ 2017)
Standard of care						
Only full clinic visits (Standard of care full)	$7.73	6	0	0	0	$46
Mix of full and short clinic visits (Standard of care short)	$2.73	2	4	0	0	$26
Decentralized medication delivery						
Only 2 months fee^b^ (DMD fee only)	$3.59	2	0	4	0	$30
2 months fee + support (DMD fee plus support)	$6.32	2	0	4	0	$41
Adherence clubs						
Base case (AC base)	$6.67	2	0	0	4	$42
Visits cost 50% less (AC lower limit)	$3.34	2	0	0	4	$29
Visits cost 50% more (AC lower limit)	$10.01	2	0	0	4	$56

^a^ Based on guidelines

^b^ Fee based on the Centralised Chronic Medicines Dispensing and Distribution (CCMDD) Programme.

### 2.8 Question 8: Should differentiated care for stable HIV patients in South Africa increase or decrease costs of HIV care and treatment?

In Table 4, we estimate the total cost of treatment per patient per year for each model based on the guideline quantities of resource utilization. Because ARV medications and laboratory tests jointly comprise such a large share of treatment costs, the variation in total cost among the models is modest, ranging from $169/patient/year to $199/patient/year. We speculate that the most realistic estimates are those for SOC short ($169), DMD fee plus support ($184) and AC base case ($185), so it appears likely that both differentiated models of care considered here will cost very slightly more in terms of provider costs than SOC, on average.

**Table 4:** Cost/patient/year by model, using guideline‐based resource quantities (US $2017)

Component	Standard of care (SOC) full	SOC short	Decentralized medication delivery (DMD) fee only	DMD fee plus support	Adherence Club (AC) base	AC lower limit	AC upper limit
Visit costs^a^	46	26	30	41	42	29	56
ARV costs	119	119	119	119	119	119	119
Laboratory costs (1 viral load)	24	24	24	24	24	24	24
Total per year	189	169	173	184	185	172	199

Table 4 can also be used to consider implications of other possible approaches to differentiated care not included in the adherence guidelines. For example two annual clinic visits (full visits), with six6‐month drug refills would reduce visit costs to just $15.46 (somewhat lower than the SOC short model in Tables 3 and 4. Such alternative models were not, however, included in the adherence guidelines or AGL evaluation.

## 3 CONCLUSIONS

Alternative models of care for treatment‐experienced, stable patients, such as AC and DMD, are designed to make it easier and less costly (time and money) for these patients to collect medications, adhere to medications, and remain virally suppressed [28]. Provided these approaches are delivered outside of the clinic, and assuming that a high proportion of clinic patients are stable on treatment and choose to receive their medications through these differentiated models of care, these models have the potential to ease crowding at facilities, reduce waiting times and possibly improve the quality of care for those patients who remain at the clinic. Achieving these benefits assumes that facilities are sufficiently staffed prior to patients being referred into well‐functioning AC and DMD models and depends on the effective use and reassignment of clinic resources and staff time that were previously used to treat those stable patients.

In reality, some clinics already manage stable patients efficiently in their standard, clinic‐based model of care. For these clinics, cost reductions through alternative models of care might be limited, but reducing visits to the clinic might generate benefits to stable patients (e.g. potentially less waiting time at the clinic) and other patients (e.g. through possible clinic decongestion). Clinics that are able to manage AC at lower cost/patient, for example through larger groups and/or shorter group visits, but otherwise require “full” clinic visits for their clinic‐based care patients, can achieve cost reductions through AC, though they may also be able to achieve cost reductions through better management of clinic‐based care.

While recent models predict reductions in the costs of care and treatment with differentiated care compared to conventional care [16], possible cost reductions for stable patients in such models may be driven by comparing a rather onerous undifferentiated, conventional model of care to more streamlined‐differentiated models. By 2019 in South Africa, substantial differentiation of service delivery had already taken place, making further significant reductions in annual budgetary needs for stable patients unlikely, with the possible exception of introducing multi‐month dispensing. This may or may not the be the case in other countries, depending on what constitutes “standard of care” at any given timepoint. While our methods are widely applicable, the results of our analysis pertain to South Africa at a particular moment in time, and it underscores the importance of both having and describing a standard‐of‐care comparison when reporting the outcomes of DSD models.

Cost‐saving opportunities for DSD models may be limited by the “lumpiness” of healthcare system resources. There will be no reduction in costs if, for example DSD models lead to fewer visits to nurses but do not affect the use of nurses’ time or salary costs, as they must be paid full time wages regardless. There is an expectation that both staff time and clinic space will be “freed up” by DSD models, allowing the same clinic infrastructure and staff complement to manage more patients (HIV and otherwise), invest more consultation time on struggling ART patients, provide higher quality care, improve other aspects of clinic management, such as record‐keeping, or undertake more outreach services outside the facility. Achieving these benefits assumes the efficient re‐allocation and use of clinic resources, including staff time, that were previously used to treat stable patients under the conventional model of care. To date, there are few if any data to document how clinics are using their newly available resources, or if such resources exist at all.

Finally, as mentioned earlier, cost savings to providers are not the only, and perhaps not even the most important, benefit sought by countries that are scaling up DSD models. For policy makers and programme managers, savings to patients, due to lower transport fares, fewer hours spent waiting in queues, and more convenient interactions with the healthcare system overall, may well outweigh changes to provider costs as the primary consequence of DSD. In the context of the COVID‐19 response, DSD models that emphasize ease of medication pickups outside of facilities and with longer refill quantities, which aid social distancing, could be essential for effectively supporting continued, high‐quality care for patients.

While patients’ benefits and costs are beyond the scope of this article, we conclude by acknowledging their importance to the DSD model debate, regardless of whether provider costs increase or decrease.

## COMPETING INTERESTS

None to declare.

## AUTHORS’ CONTRIBUTIONS

BAL contributed to the discussion of manuscript and research design, managed and analysed the data and drafted and revised the manuscript. SJSP contributed to the discussion of manuscript and research design, developed the primary data, supported the data management and analysis and drafted and revised the manuscript. AH contributed to the research design, developed the primary data, supported the data management and analysis and drafted and revised the manuscript. LCL contributed to the research design, supported the development of unit costs and revised the manuscript. JM contributed to the research design, supported the data development and revised the manuscript. SBR contributed to the discussion of manuscript design, designed the research, advised on the data analysis and drafted and revised the manuscript. MPF designed the research, advised on the data analysis and drafted and revised the manuscript. NFH contributed to the research design, advised on the data analysis and revised the manuscript.
